# The Synthetic Tryptanthrin Analogue Suppresses STAT3 Signaling and Induces Caspase Dependent Apoptosis via ERK Up Regulation in Human Leukemia HL-60 Cells

**DOI:** 10.1371/journal.pone.0110411

**Published:** 2014-11-10

**Authors:** Anup S. Pathania, Suresh Kumar, Santosh K. Guru, Shashi Bhushan, Parduman R. Sharma, Sravan K. Aithagani, Parvinder P. Singh, Ram A. Vishwakarma, Ajay Kumar, Fayaz Malik

**Affiliations:** 1 Department of Cancer Pharmacology, Indian Institute of Integrative Medicine, Jammu and Kashmir, India; 2 Medicinal chemistry division, Indian institute of Integrative Medicine, Jammu and Kashmir, India; 3 Academy of Scientific and Innovative Research (AcSIR), New Delhi, India; 4 Experimental Breast Cancer Research Laboratory, University of Michigan North Campus Research Complex, Ann Arbor, Michigan, United States of America; Wayne State University School of Medicine, United States of America

## Abstract

Tryptanthrin is a natural product which has been reported to have several medicinal properties. In this study, we tried to investigate the detailed molecular mechanism of its bromo analogue (TBr), a potent cytotoxic agent in the induction of cancer cell death. It was found that TBr primarily targets STAT3 and ERK signaling during the induction of apoptosis in several human leukemia cell lines. In HL-60 cells, TBr treatment caused early down regulation of p-STAT3 with concomitant up regulation of p-ERK which led to the activation of intrinsic and extrinsic pathways of apoptosis. The mechanism of TBr mediated inhibition of p-STAT3 was found to be due to the activation of ubiquitin dependent degradation of tyrosine 705 and serine 727 p-STAT3. As IL-6 is the main driver of the STAT3 pathway, the effect of TBr on cell death was subdued when treated in the combination with IL-6 in HL60 cells. Interestingly, PD98059 significantly reduced the apoptotic effects of TBr, thus showing the direct involvement of p-ERK in TBr mediated cell death. It was further shown that apoptotic protein Bax silencing in HL-60 cells resists TBr mediated ERK dependent apoptosis. In summary, for the first time we report the mechanism of TBr mediated cell death in human leukemia cell lines by targeting STAT3 and ERK pathways.

## Introduction

STATs or Signal Transducers and Activators of Transcription control growth, survival and differentiation in cancer cells. Dysregulation of STATs signaling is frequently observed in leukemia cells that lead to an increase in their proliferation, growth and uncontrolled division [1], [2]. STATs are activated by cell surface receptors mainly cytokine receptors via phosphorylation at its tyrosine and serine residues catalyzed by Jak family kinases, intrinsic receptor tyrosine kinases and other cellular tyrosine kinases such as c-Src. Once phosphorylated, STAT proteins form dimers and translocate to the nucleus where it acts as transcription factors for many genes involved in cellular proliferation. Constitutive activation of STAT1, STAT3 and STAT5 have been demonstrated in both acute and chronic leukemia [3] and STATs activation alone has been shown to cause cellular transformation in certain cellular backgrounds [4]. AML or Acute myeloid leukemia is a cancer of the myeloid line of blood cells, characterized by the rapid growth and accumulation of white blood cells in the bone marrow, which interferes with the production of normal blood cells. AML can occur at any age but is more common in adults over the age of 60. AML is mainly treated by chemotherapy, and natural products play an important role in the treatment of these hematological malignancies [5], [6], [7]. Many of the current drugs used in the treatment of leukemia are from natural products like vinca alkaloids and their derivatives, podophyllotoxin derivatives, indirubin, flavopiridol and various others are currently undergoing preclinical investigations.

Tryptanthrin (6, 12-dihydro-6, 12-dioxoindolo-(2, 1-b)-quinazoline) is a natural alkaloid found in many plant species [8]. Earlier studies have reported various biological and pharmacological activities of tryptanthrin including anti-inflammatory [9], anti-microbial [10], anti-trypanosomal [11] and immunomodulatory [12], [13]. In recent years, tryptanthrin has gained much attention as an anticancer agent [14], [15], [16] but its biology in cancer cells remains unexplored. In this study, we have used a more potent analog of tryptanthrin (tryptanthrin bromo or TBr) to investigate the underlying molecular mechanism of its anti-cancer activity in leukemia cells. We are showing for the first time that TBr blocked STATs signaling and induced caspase dependent apoptosis in leukemia cells. Furthermore, in depth study in human leukemia HL-60 cell line showed that TBr induced cell death involved ubiquitin dependent degradation of p-STAT3 with subsequent increase in p-ERK expression. We further demonstrated that p-ERK up regulation by TBr promoted apoptosis in HL-60 cells and this is accompanied by Bax upegulation.

## Materials and Methods

RPMI-1640, propidium iodide (PI), rhodamine-123, 3-(4, 5, -dimethylthiazole-2-yl)-2, 5 diphenyltetrazolium bromide (MTT), penicillin, streptomycin, fetal bovine serum, L-glutamine, pyruvic acid, MG132, IL-6, protease inhibitor cocktail and sodium fluoride were purchased from Sigma-Aldrich (St Louis, MO). MEK1/2 inhibitor PD98059 (PD) and U0126 were purchased from Calbiochem (Gibbstown, NJ). AnnexinV-FITC apoptosis detection kit were purchased from B.D Biosciences (San Jose, CA). Anti-human antibodies were purchased from Santa Cruz Biotechnology (Santa Cruz, CA) and Cell Signaling Technology (Danvers, MA). Pan caspase inhibitor Z-VAD-fmk, transfection reagent, transfection medium and Bax siRNA were purchased from Santa Cruz Biotechnology. Electrophoresis reagents, reagents for protein estimation and protein molecular weight markers were from Bio-Rad Laboratories (Hercules, CA).

### Cell Culture conditions

Human leukemia cell lines HL-60, MOLT-4, K-562 and normal breast epithelial cell line fR2 were obtained from ECACC, England. Cells were grown in RPMI medium containing 10% FBS and 1X antibiotic solution. Cells were grown in a CO_2_ incubator at 37°C with 95% humidity and 5% CO_2_.

### Synthesis and structure of TBr

TBr was prepared by the condensation of isatoic anhydrides with isatins in toluene by refluxing in the presence of triethylamine. After cooling, the reaction mixture was evaporated in using a vacuum and crude mixtures were purified through column chromatography. Pure compounds were characterized through NMR and mass spectroscopy. The structure of the parent compound tryptanthrin and TBr are given in Fig. S1.

### Cell Proliferation Assay

The antiproliferative effect of TBr was determined by MTT assay. Cells were seeded in 96 well plates and exposed to different concentrations of TBr. ERK inhibitor PD98059 or IL-6 were added 1 h before TBr addition. MTT dye (50 µg/ml) was added 3 h before experiment termination and MTT formazen crystals were dissolved in DMSO. OD was taken at 570 nm spectrophotometrically.

### Cell cycle analysis

Cells were treated with TBr at different doses and time intervals. Inhibitors used in this study were added 1 h before TBr addition. After treatment, cells were fixed in 70% alcohol overnight at 4°C. Next day, cells were digested with RNase (200 µg/ml) at 37°C for 1 h followed by PI (10 µg/ml) incubation for another 30 min. Cells were acquired in FACS Calibur (BD, San Jose, CA) for cell cycle phase distribution and the data was analyzed by ModFit software (Verity Software House Inc., Topsham, ME).

### Mitochondrial membrane potential

Mitochondrial membrane potential (MMP) in cells was analyzed by using a fluorescent probe rhodamine-123, added to the cells 40 min before the termination of the experiment. Cells were collected, washed in PBS twice and the fluorescence intensity from 10,000 events was analyzed in the FL-1 channel of flow cytometer.

### Whole cell lysates preparation for immunoblotting

TBr treated and untreated cells were lysed in cold lysis buffer (RIPA) containing 50 mM NaF, 0.5 mM NaVO_4,_ 2 mM PMSF and 1% protease inhibitor cocktail for 40 min. Cells were centrifuged at 12000×g for 10 min at 4°C and the supernatant was collected as whole cell lysates.

### Preparation of mitochondrial and cytosolic fraction for immunoblotting

For cytosolic fraction preparation, cells were lysed with cell lysis buffer (75 mM NaCl, 8 mM Na_2_HPO_4_, 1 mM NaH_2_PO_4_, 1 mM EDTA, and 350 µg/ml digitonin and 1% (v/v) eukaryotic protease inhibitor cocktail) for 1–2 min followed by centrifugation at 12,000×g for 2 min at 4°C. The supernatant were collected as cytosolic fraction and the residual pellet were lysed with RIPA buffer for 30 min on ice. Lysates were centrifuged at 12,000×g for 10 min and the supernatant was taken as mitochondrial fraction.

### Western blot analysis

Protein content was measured by Bradford reagent (Bio-Rad) and 30–70 µg protein amount were run by using SDS-PAGE followed by western blotting as described earlier [17].

### Immunofluorescence

Immunofluorescent staining for p-STAT3 in TBr treated and untreated cells were performed by using fluorescence microscopy. After treatment, cells were transferred to polylysine coated cover slips and fixed in 4% paraformaldehyde/0.1% Triton-X100 solution. Cells were probed with anti p-STAT3 antibody (Y705) antibody overnight followed by incubation with alexa fluor 555 conjugated anti-rabbit secondary antibody (Cell signaling technology) for 1 h. The cover slips were counterstained with 4, 6-diamidino-2-phenylindole (DAPI) to mark the nuclei and photographs were taken.

### Immunoprecipitation

Control and treated cells were lysed in RIPA buffer for 5–10 min followed by heating at 95°C for 5 min. The suspension was diluted with non-denaturing lysis buffer (20 mM Tris HCl, pH 8, 137 mM NaCl, 1% Nonindet P-40 (NP-40), 2 mM EDTA and 1% protease inhibitor cocktail) and incubated on ice for 10 min. Protein content was measured by Bradford reagent and the samples containing 250 µg of protein were incubated with p-STAT3 (Y705) antibody overnight at 4°C followed by incubation with protein A/G-agarose beads for another 6 h. After incubation, samples were centrifuged 3 times and supernatant was removed after every step. Finally 25 µl 2X loading buffer was added to pellet followed by boiling at 100°C for 5 min to denature proteins and separate it from beads. Samples were centrifuged and the supernatant was used for western blotting.

### Gene Silencing with siRNA

siRNA transfection was performed by using lipofectamine reagent from Santa Cruz Biotechnology according to the manufacturer’s instructions. Briefly, 1×10^6^ cells were seeded in 3 ml of transfection medium containing 50 nM Bax siRNA for 8 h. After incubation, transfection media was replaced with RPMI containing 2X fetal bovine serum and 2X antibiotic solution. Cells were harvested for experimental purposes during 24–72 h.

### Statistical Analysis

Data is presented as means of two or three similar experiments and the error bars represent the standard deviation (SD) between the experiments. Statistical analysis was done by using the Bonferroni method and p value<0.05 was considered to be significant with ***p<0.001, **p<0.01, *p<0.05.

## Results

### TBr demonstrated antiproliferative activity in leukemia cell lines

**Figure 1:**
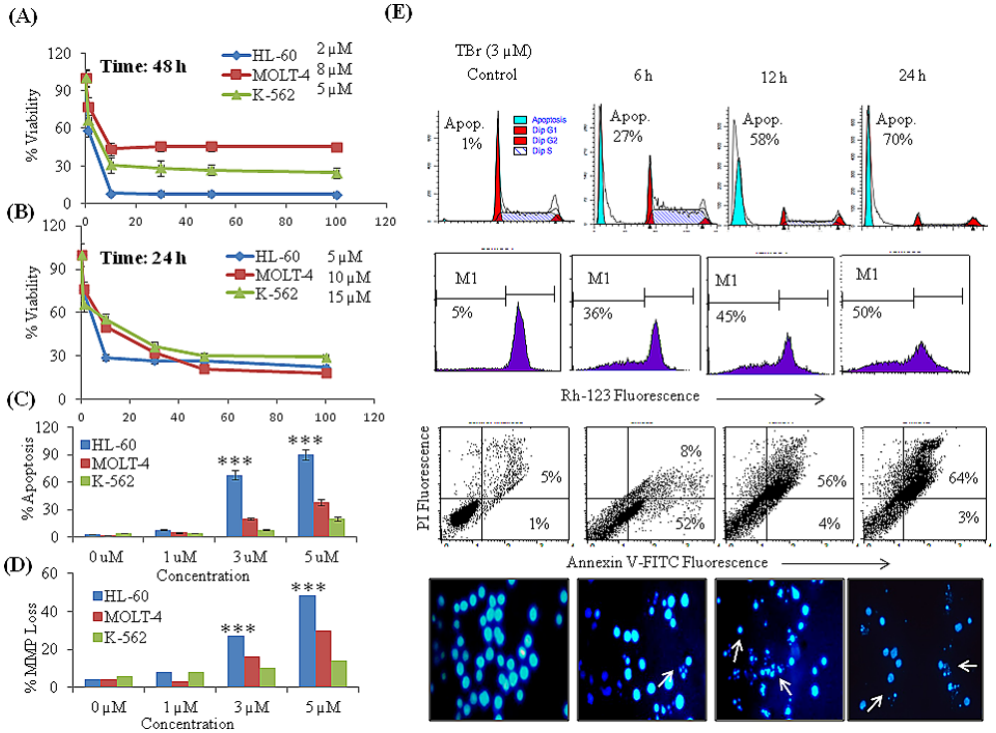
TBr inhibited cell proliferation in leukemia cell lines. (A–B) Cell proliferation assay. Cells were treated with 1, 10, 30, 50 and 100 µM concentrations of TBr for 24 and 48 h. Cells were subjected to MTT assay to analyze cell proliferation. (C) DNA cell cycle analysis for subG1 population. Cells were treated with TBr for 24 h at indicated concentrations and stained with PI to determine DNA Fluorescence. SubG1 DNA population having <2n DNA were analyzed by ModFit software. (D) Mitochondrial membrane potential loss. Cells were treated with various concentrations of TBr for 24 h and stained with rhodamine-123. MMP loss was analyzed by flow cytometry and a decrease in rhodamine-123 fluorescence was taken as indicative of MMP loss. (E) TBr (3 µM) increased subG1 population, triggered MMP Loss, increased annexin V positive apoptotic cells and induced apoptotic bodies’ formation in HL-60 cells. Data are mean ± SD of three similar experiments and statistical comparisons of TBr treated HL-60 vs MOLT-4 and K-562 cells were made by using the Bonferroni method, p value<0.05 was considered significant, ***p<0.001.

The cytotoxicity of TBr was evaluated in various leukemia cell lines including HL-60, MOLT-4 and K-562. TBr treatment inhibited the proliferation of leukemia cell lines in dose and time dependent manner. IC50 values of TBr in HL-60, MOLT-4 and K-562 cell lines were 5, 10 and 15 µM for 24 h and 2, 8 and 5 µM for 48 h respectively (Fig. 1A and 1B). On the contrary, the cytotoxicity of the parent compound tryptanthrin in HL-60 cells was found to be almost four times higher with an IC50 value of 20 µM in 48 h (Data not shown). Additionally, it required more than five times higher concentration of TBr to induce comparable cytotoxicity in normal breast epithelial cell line fR2 in 48 h (Supporting Fig. 2A). FACS analysis showed that TBr increased subG1 DNA population and induced MMP loss in all three leukemia cell lines in a dose dependent manner (Fig. 1C and 1D). However, TBr treated fR2 cells demonstrated a substantial reduction in the DNA damaging effect (Fig. S2B). On the basis of these results we chose HL-60 cells for further detailed studies. Time dependent cell cycle analysis of TBr treated HL-60 cells revealed the appearance of 27% of the cells under subG1 peak in 6 h which was increased to 70% through 24 h (Fig. 1E). Similar treatments with TBr induced 36%, 45% and 50% MMP Loss in HL-60 cells at indicated time intervals. The presence of apoptotic bodies observed in cells stained with hoechst after treatment with TBr further confirmed that TBr triggered cell death by apoptosis (Fig. 1E).

**Figure 2:**
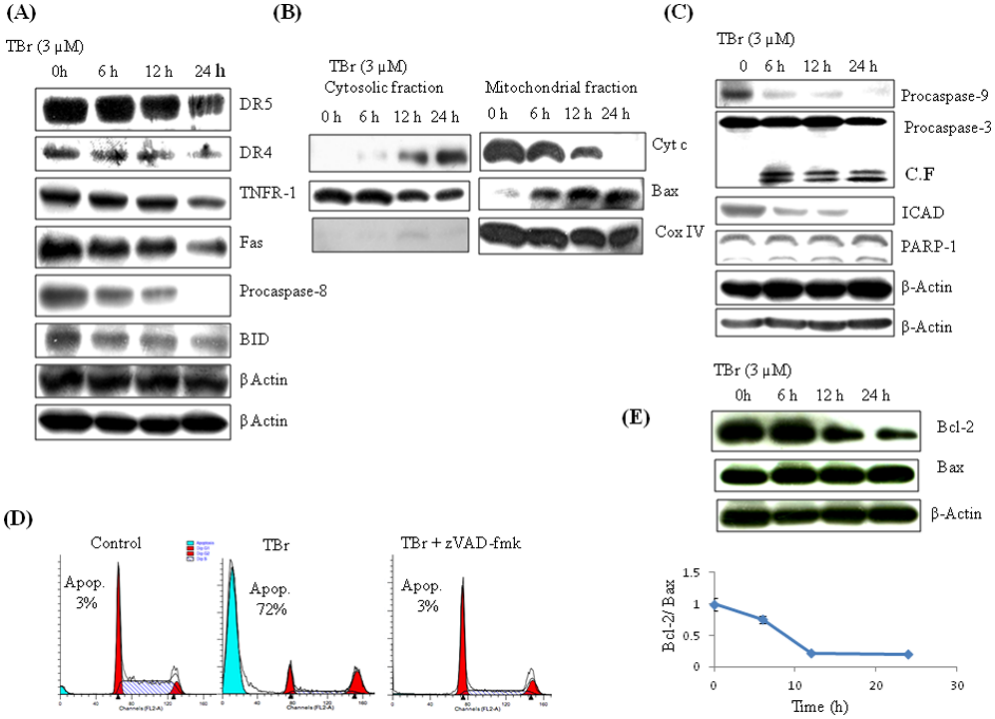
TBr induced apoptosis in HL-60 cells involves both intrinsic and extrinsic apoptotic pathways. (A) TBr caused the activation of caspase-8 and its downstream target Bid independent of death receptors. HL-60 cells were treated with TBr at 3 µM concentration for indicated time periods. Cells were collected and protein lysates were prepared for imunoblotting of indicative proteins as described in material and methods section. β actin was used as a loading control. (B) TBr treatment triggered the cytosolic translocation of Cyt c from the mitochondria, and mitochondrial translocation of Bax from cytosol. CoxIV was used as a control for mitochondrial fraction purity. (C) TBr caused the activation of caspase-9 followed by caspase-3 and PARP-1 cleavage. β actin was used as an internal control. (D) Cell cycle analysis of TBr (3 µM) treated cells for 24 h along with pan caspase inhibitor z-VAD-fmk (30 µM). Caspase inhibitor was added 1 h before TBr treatment. Cells were collected and stained with PI to determine DNA fluorescence by flow cytometery. (E) Bcl-2/Bax ratio in TBr treated cells in a time dependent manner.

### TBr induced cell death in human leukemia HL-60 cells is caspase dependent

Caspases are the key mediators of apoptosis. TBr triggered the activation of both intrinsic and extrinsic pathways of apoptosis in HL-60 cells. TBr induced time dependent activation of caspase-8 without the involvement of death receptors TNFR-1, FasR, DR4 and DR5 (Fig. 2A). Activation of caspase-8 was also evident by the cleavage of its downstream target protein Bid (Fig. 2A). Further experiments showed that TBr could trigger the release of cytochrome c from the mitochondria after the translocation of Bax from the cytosol (Fig. 2B). In addition to the release of cytochrome c, TBr also caused the time dependent activation of caspase-9, leading to further activation of executioner caspase-3. This was also evident by the cleavage of its downstream target PARP-1 (Fig. 2C). However, inhibition of caspases by pre-treatment with pan caspase inhibitor Z-VAD-fmk led to the complete reversal of TBr-induced apoptosis, as indicated by the disappearance of the subG1 population (Fig. 2D). The strong apoptotic effect of TBr was further demonstrated by the drastic reduction in the Bcl-2/Bax ratio in a time dependent manner in HL-60 cells (Fig. 2E).

### TBr suppressed STATs signaling in leukemia cell lines

**Figure 3:**
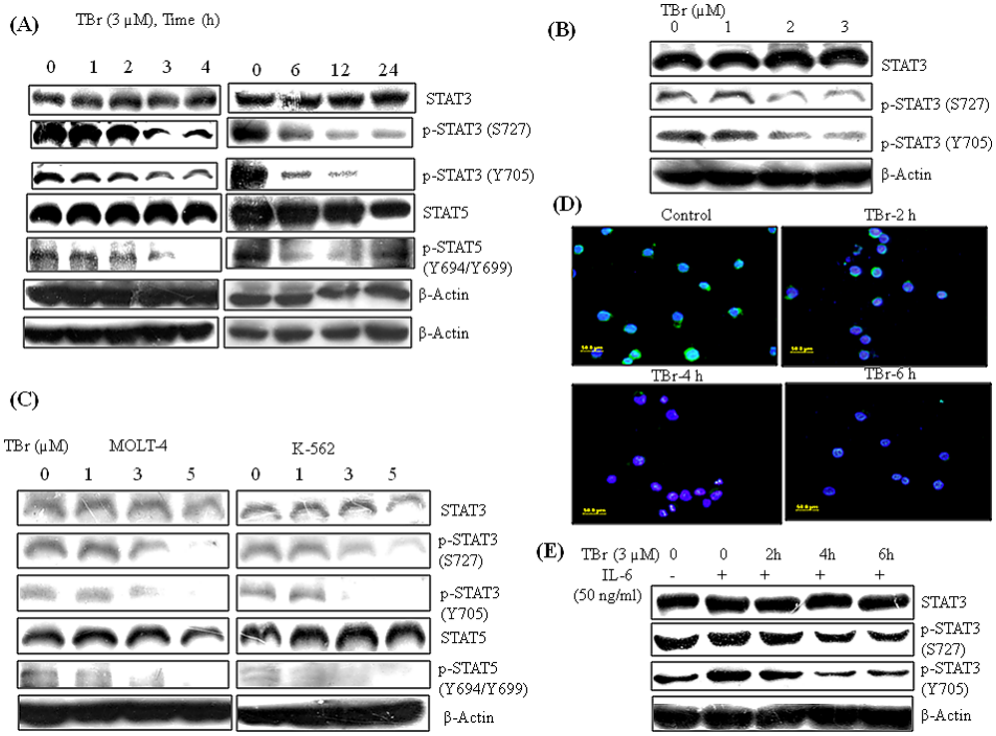
TBr inhibited STATs signaling in leukemia cell lines. (A) Cells were treated with TBr (3 µM) at indicated time intervals and protein lystaes were prepared for immunoblotting of indicative proteins (B) Concentration dependent inhibition of p-STAT3 (Y705 and S727) in TBr treated HL-60 cells for 6 h. (C) TBr inhibited STATs signaling in MOLT-4 and K-562 cells, when treated for 6 h at indicated concentrations. β actin was used as a loading control. (D) Immunofluorescence of p-STAT3 (Y705) in TBr (3 µM) treated HL-60 cells. TBr treatment significantly decreased p-STAT3 (Y705) expression as indicated by the decrease in green fluorescence. (E) TBr inhibited IL-6 induced STAT3 phosphorylation. HL-60 cells were pre-treated with TBr (3 µM) at indicated time intervals and IL-6 (50 ng/ml) was added 30 min before experiment termination to induce p-STAT3 expression.

STATs are reported to be constitutively activated in leukemia cell lines and are involved in cell growth and proliferation (1, 2). Aberrant activation of STAT3 is frequently associated with aggressiveness of the disease and is activated by phosphorylation at tyrosine 705 and serine 727 residues, which induces its dimerization, nuclear translocation, and DNA binding [18]. The effect of TBr on STATs signaling was thus explored in HL-60 cells over a range of time points from 1 to 24 h. TBr showed a strong inhibitory effect on the phosphorylation of STAT3 at tyrosine 705 and serine 727 residues in a time and concentration dependent manner (Fig. 3A & B). A similar effect of TBr was observed in the case of STAT5, where the decreased expression of p-STAT5 (Y694/Y699) was observed even at an early time point of 3 h (Fig. 3A). Early time treatments with TBr also demonstrated a clear inhibitory effect on the expression of phosphorylated STAT3 and STAT5 in other leukemia cell lines MOLT-4 and K-562 (Fig. 3C). Immunofluorescent staining further confirmed the inhibitory effect of TBr on p-STAT3 (Y705) in a time dependent manner, where fluorescence microscopy images showed a significant suppression of p-STAT3 (Y705) in HL-60 cells (Fig. 3D). Furthermore, TBr was also able to suppress the IL-6 induced phosphorylation of STAT3 (Y705 and S727) in human leukemia HL-60 and K-562 cells (Fig. 3E and Fig. S3).

### TBr down regulates STAT3 target genes and promotes ubiquitination of activated STAT3

**Figure 4:**
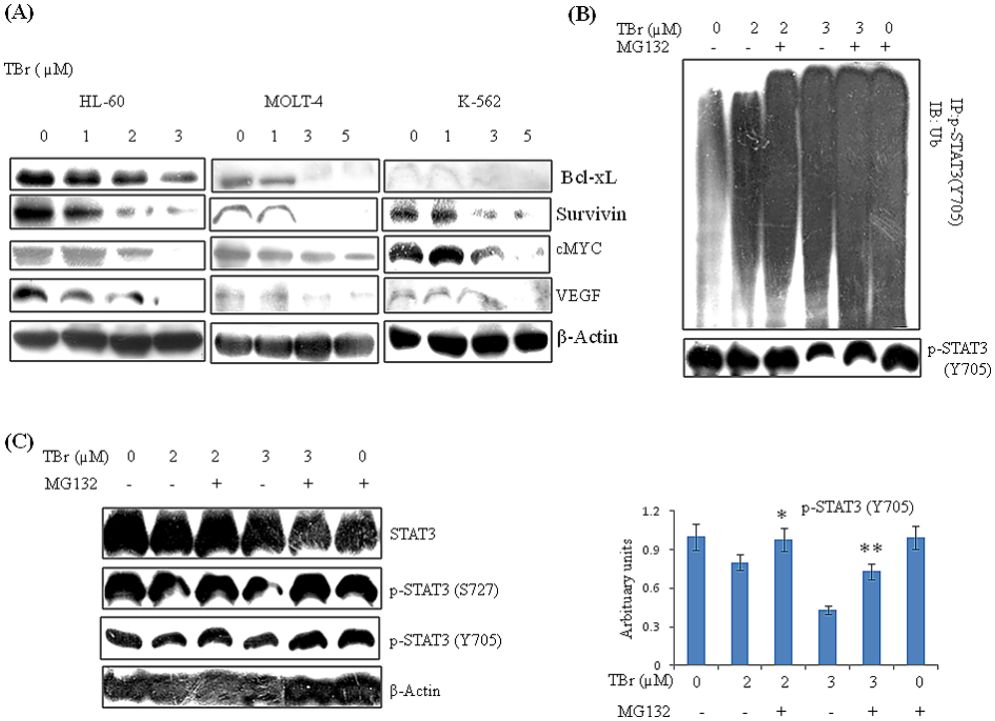
TBr down regulated STAT3 target genes and promoted ubiquitin dependent degradation of activated STAT3. (A) HL-60, MOLT-4 and K-562 cells were treated with TBr at indicated concentrations for 6 h. Lysates were prepared and immunoblotting of indicated proteins were performed. β actin was used as the loading control. (B) HL-60 cells were treated with TBr at 2 and 3 µM concentrations along with the proteasomal inhibitor MG132 (0.07 µM) for 6 h. Protein lysates were prepared and p-STAT3 (Y705) was immunoprecipitated with its respective antibody. Immunoblotting was used to detect antiubiquitin (P4D1, Santacruz) and p-STAT3 (Y705) antibodies. A predominant ubiquitination of the tyrosine705 phosphorylated STAT3 is seen in the TBr treated cells under proteasomal inhibition. (C) MG132 pre-treatment rescued decreased p-STAT3 expression in TBr treated HL-60 cells. Data are mean ± SD of three similar experiments, *p<0.05, **p<0.01 for TBr vs TBr with MG132.

Our initial results showed that STAT3 is a key target of TBr-induced cytotoxicity. In order to get further insight on the mechanism of TBr, we checked the expression of STAT3 target genes. Interestingly, the expression of several tumorigenic genes such as Bcl-xL, survivin, myc, and VEGF were strongly decreased even at early time points in all the three leukemia cell lines HL-60, MOLT-4 and K-562 (Fig. 4A). We further questioned if there is any effect of TBr on proteasomal regulation of STAT3. To address this question, we treated the HL-60 cells with proteasomal inhibitor MG132 in the presence or absence of TBr and immune-precipitated p-STAT3 (Y705) before running the western blot for ubiquitin and p-STAT3 (Y705). The results clearly indicated that TBr even at a low concentration of 2 µM was able to induce ubiquitination of p-STAT3 (Y705) and when MG132 was added, the level of ubiqitination increased due to accumulation of ubiquitinated pSTAT3 (Y705) (Fig. 4B). Similar results were observed with the p-STAT3 (Y705) antibody, where MG132 increased the expression of p-STAT3 (Y705) (Fig. 4B). Western blot analysis for phosphorylated STAT3 (Y705 and S727) in a whole cell lysates of HL-60 cells treated with TBr and MG132 also displayed similar results (Fig. 4C).

### TBr promotes ERK mediated apoptosis in human leukemia HL-60 cells

**Figure 5:**
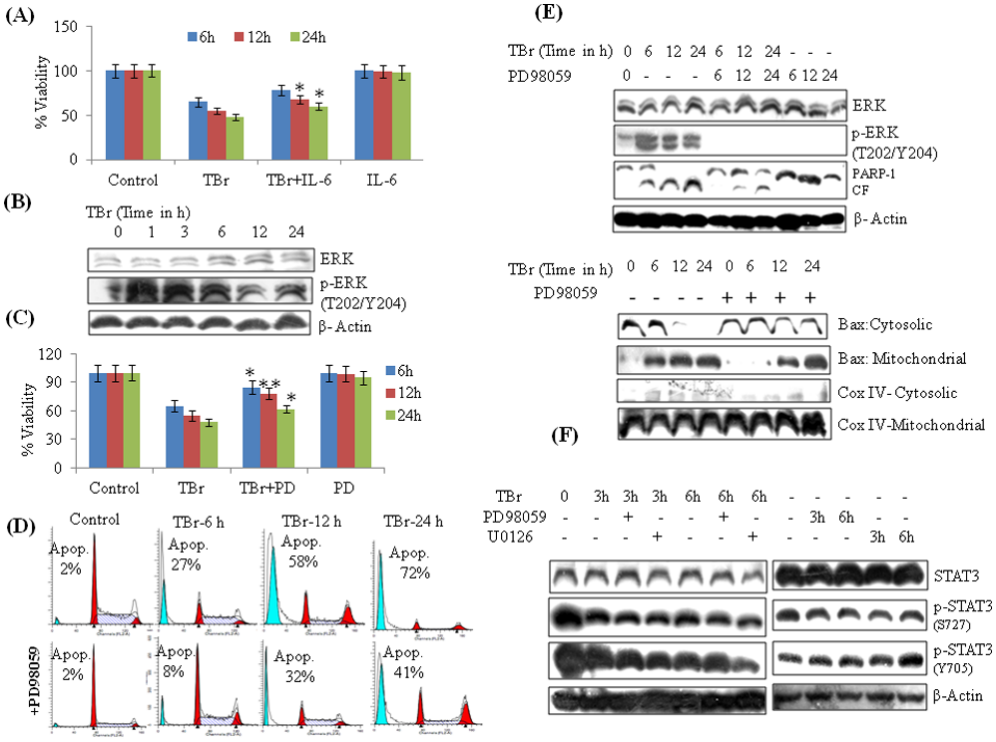
TBr induced p-ERK dependent apoptosis in HL-60 cells. (A) IL-6 treatment increased cell viability in TBr treated cells. Cells were pre-treated with IL-6 (50 ng/ml) for 40 min followed by TBr (3 µM) addition for another 24 h. MTT dye was added 3 h before the experiment termination. Data are mean ± SD from three experiments. (B) TBr (3 µM) induced p-ERK expression in HL-60 cells (C) MTT assay of TBr treated cells along with ERK inhibitor PD98059 (30 µM) at indicated time periods. PD98059 was added 1 h before TBr addition. (D) Effect of PD98059 on subG1 population in TBr treated cells. After treatment, cells were stained with PI and DNA fluorescence was determined by flow cytometery. (E) Effect of PD98059 on Bax and PARP-1 cleavage in TBr treated HL-60 cells. Cells were treated with TBr (3 µM) and PD98059 (30 µM) at indicated time intervals. Whole, mitochondrial and cytosolic protein lystaes were prepared and equal amount of proteins were loaded and resolved on SDS PAGE followed by immunoblotting as described in material and methods section. (F) Effect of PD98059 (10 µM) and U0126 (10 µM) on STAT3 expression in TBr treated cells. β actin was used as a positive control. Data are mean ± SD of three similar experiments and statistical comparisons of TBr vs. TBr with PD98059/IL-6, was made by using the Bonferroni method, *p<0.05, **p<0.01.

Our data showed that STAT3 played an important role in the TBr-induced cytotoxicity, although induction of STAT3 by IL-6 in TBr treated cells could only partially rescue the cells from cytotoxicity (Fig. 5A). Therefore, we reasoned that other crucial pathways might be playing a role in this process. We then focused our attention on the ERK signaling pathway, which plays an important role in cellular proliferation and growth; however its over-activation may lead to apoptosis by triggering intrinsic and extrinsic pathways [19]. Interestingly, TBr treatment in HL-60 cells resulted in a sharp increase in the expression of p-ERK (T202/Y204), even after 1 h of exposure (Fig. 5B). In order to assess the role of ERK in TBr mediated apoptosis, we inhibited the ERK pathway by pre-treatment with pharmacological inhibitor PD98059. Surprisingly, TBr-induced cell cytotoxicity and DNA damage were significantly reversed on PD98059 pre-treatment in a time dependent manner (Fig. 5C & D). Pre-treatment with PD98059 also reversed the TBr effect on PARP-1 cleavage and mitochondrial translocation of proapoptotic Bax (Fig. 5E). HL-60 cells treated only with PD98059 showed no caspase-3 and PARP-1 cleavage (Supporting Fig. 4). Taking a cue from the literature, where ERK is reported negatively regulating STAT3 transcriptional activities in some cells [20], [21], we further tried to elucidate any cross talk between these pathways. We suppressed the ERK pathway by pharmacological inhibitors PD98059 and U0126 before analyzing the expression of STAT3 in TBr treated HL-60 cells. However, the inhibition of ERK by any of the inhibitors was not able to rescue the decreased expression of STAT3 in TBr treated cells (Fig. 5F).

### ERK mediated apoptotic induction was accompanied by Bax up regulation

**Figure 6:**
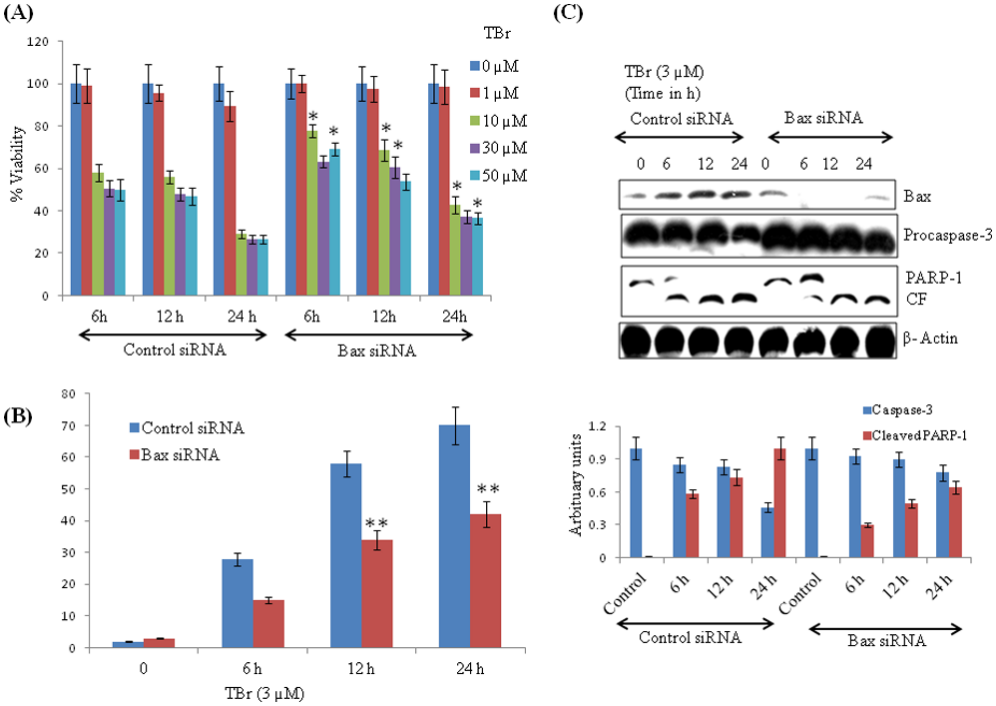
Bax silencing rescued the TBr-mediated cytotoxic effect in HL-60 cells. (A) Cells containing control and Bax siRNA were treated with TBr at indicated concentrations for 24 h and MTT assay was performed. (B) Cell cycle analysis of TBr treated Bax positive and Bax silenced cells. (C) PARP-1 and Caspase-3 expression in Bax positive and Bax silenced TBr treated cells. β actin was used as a positive control. Data are mean ± SD of three similar experiments, *p<0.05, **p<0.01 for control vs Bax siRNA.

As observed in the Figure 5E, ERK inhibition reversed the mitochondrial translocation of Bax, and we further tried to explore the role of Bax in TBr mediated ERK-induced apoptosis. Interestingly, silencing Bax by siRNA led to a significant reversal of the cytotoxic effect of TBr on cell viability and apoptosis. The apoptotic population was reduced by about 35% in 24 h in Bax silenced cells (Fig. 6A & B). Similarly, silencing of Bax also led to a significant restoration of function of caspase-3 and subsequent cleavage of PARP-1 in TBr treated HL-60 cells (Fig. 6C).

## Discussion

Natural products are always an attractive target for cancer drug discovery and still most of the clinically approved drugs are derived from natural products or their analogues [22], [23]. Tryptanthrin is a natural product found in many indigo plant species and has been reported having various pharmacological activities [9], [10], [11], [12]. There are some earlier reports of the cytotoxic potential of tryptanthrin and its apoptotic activity but its detailed mechanism of action is not known [24], [25]. In this current study, the underlying mechanism of tryptanthrin was investigated in human leukemia HL-60 cells. We used tryptanthrin bromo (TBr), a tryptanthrin analogue which is more potent than its parent compound against human leukemia cell lines. TBr treatment significantly suppressed cell viability in human leukemia cell lines HL-60, MOLT-4 and K-562 with IC50 values of 2, 8 and 5 µM for a 48 h. TBr treatment increased the sub-G1 apoptotic population of cells and triggered MMP loss in all three cell lines, with prominent activity in HL-60 cells. Time dependent studies in HL-60 cells further confirmed its proapoptotic potential, which is measured by several biological endpoints such as annexin V binding, hoechst staining, MMP loss and the increase in subG1 population of cells.

Death signaling induced by TBr required both intrinsic and extrinsic apoptotic pathways as evidenced by strong activation of caspase-8 and caspase-9 followed by caspase-3 cleavage in HL-60 cells. It was found that activation of caspase-8 was not associated with increased expression of apical death receptors. To further investigate the signaling events that triggered caspase-9 activation, the role of Bcl-2 protein family members and proapoptotic signaling molecules from the mitochondria were examined. Bcl-2 is constitutively activated in leukemia cells, thus creating a block in cancer cell apoptosis, which develop resistance in cells. TBr treatment decreased Bcl-2 expression and subsequently increased antiapoptotic Bax protein in a time dependent manner causing a decreased Bcl-2/Bax ratio. Additionally, TBr-induced apoptosis seems to be completely dependent on caspases, which was evident by the complete reversal of TBr-induced subG1 apoptotic population by pan caspase inhibitior z-VAD-fmk in HL-60 cells. Hence, these results indicate that apoptosis induced by TBr utilize a wide range of molecular targets that include mitochondrial dependent and independent pathways which lead to caspase-8 and -9 activation and induction of apoptosis.

The growth and progression of acute myeloid leukemia cells are supported by a constitutively high expression of STAT proteins, importantly STAT3 & STAT5 [26], [27]. TBr, even at an early time point of 2 h displayed a strong inhibitory effect on STAT3 phosphorylation at tyrosine 705 and serine 727 residues. Also, TBr substantially inhibited STAT5 phosphorylation at tyrosine 694 and tyrosine 699 residues in HL-60, MOLT-4 and K-562 cells. In order to understand the mechanism of the down regulatory effect of TBr on STAT3 signaling, we further focused on the process of ubiquitination, which is one of the mechanisms of regulation of STAT3 turnover in cells [28], [29], [30]. To this effect, we inhibited the proteasomal degradation by MG132 and analyzed the effect of TBr on the ubiquitination of STAT3. MG132 treatment led to a sharp rise in the ubiquitination of p-STAT3 (Y705) and a total reversal of the inhibitory effect of TBr on p-STAT3. This demonstrated the role of proteasomal degradation in TBr mediated inhibition of p-STAT3. Furthermore, TBr also inhibited the expression of STAT3 downstream targets such as BcL-xL, Survivin, cMYC and VEGF. The inhibitory effect of TBr on the activation of STAT3 was further supported when TBr was even able to suppress the activation of STAT3 induced by externally applied IL-6. Further experiments showed that IL-6 treatment in HL-60 cells rescued TBr mediated cytotoxicty to some extent but not fully. These results suggested that other pathways might be involved in the TBr-induced cytotoxicity.

Several earlier studies have reported that crosstalk between JAK/STAT and MAP Kinase pathways plays an important role in cancer cells growth and proliferation [31], [32]. Up regulation of ERK and PI3Kinase signaling is also reported to down regulate STAT3 activation [33]. We hypothesized that the ERK pathway may also be involved in the cytotoxicity induced by TBr. HL-60 cells treated with TBr led to a sharp increase in the expression of p-ERK1/2 (T202/Y204). Furthermore, the inhibition of the ERK pathway by PD98059 significantly reduced the TBr-induced cytotoxicity, which was evident by a reduced subG1 population and reversal of PARP-1 cleavage and mitochondrial translocation of Bax. These results seemed to be consistent with several earlier studies, where increased ERK activity has been linked with the induction of cell death [34], [35]. It was also noted that ERK pathway inhibition by PD98059 and U0126 had no effect on the down regulation of p-STAT3 by TBr. This excluded the possibility that TBr-mediated ERK activation may have caused the down regulation of STAT3 activation. ERK mediated apoptosis is accompanied by the downstream activation of several apoptotic proteins including Bax [36], [37]. Our earlier data indicated that ERK may be executing its apoptotic effect by activation of Bax. Therefore, we tried to explore the possible involvement of Bax in the TBr-induced cytotoxicity in HL-60 cells. Silencing of Bax led to a significant reversal of several cytotoxicity related end points, including cell viability, subG1 population, activation of caspase-3 and cleavage of PARP-1.

In summary, our data suggests that TBr-induced cytotoxicity in HL-60 cells requires two independent events: activation of Bax through the ERK pathway, and down regulation of STAT signaling. This study has attempted to elucidate the mechanism of TBr-induced cytotoxicity in HL-60 cells and suggests that further studies are needed to understand its potential as an anti cancer agent.

## Supporting Information

Figure S1 **Chemical structure and mode of synthesis of tryptanthrin and TBr.**
(TIF)

Figure S2 **Effect of TBr on normal human breast epithelial cell line fR2.** (A) MTT assay of TBr treated breast epithelial cell line fR2 for 48****h (B) Cell cycle analysis of TBr (3 µM) treated fR2 cell line for 24****h.
(TIF)

Figure S3 **Effect of TBr on IL-6 induced expression of p-STAT3 in the K-562 cell line.** Cells were treated with TBr at 5 µM concentration at indicated time intervals. IL-6 was added 30****min before the experiment termination to induce p-STAT3 expression. Lysates were prepared and western blotting was performed.
(TIF)

Figure S4 **Effect of PD98059 on Caspase-3 and PARP-1 cleavage in HL-60 cells.** Cells were treated with PD98059 (30 µM) for 24****h followed by protein lysates preparation, SDS PAGE and immunoblotting of Caspase-3 and PARP-1. β actin was used as a positive control.
(TIF)
