# Supplementary figures and images for: The Synthetic Tryptanthrin Analogue Suppresses STAT3 Signaling and Induces Caspase Dependent Apoptosis via ERK Up Regulation in Human Leukemia HL-60 Cells
Source: PLoS One. 2014 Nov 10;9(11):e110411. doi: 10.1371/journal.pone.0110411 (PMC4226462; doi:10.1371/journal.pone.0110411)

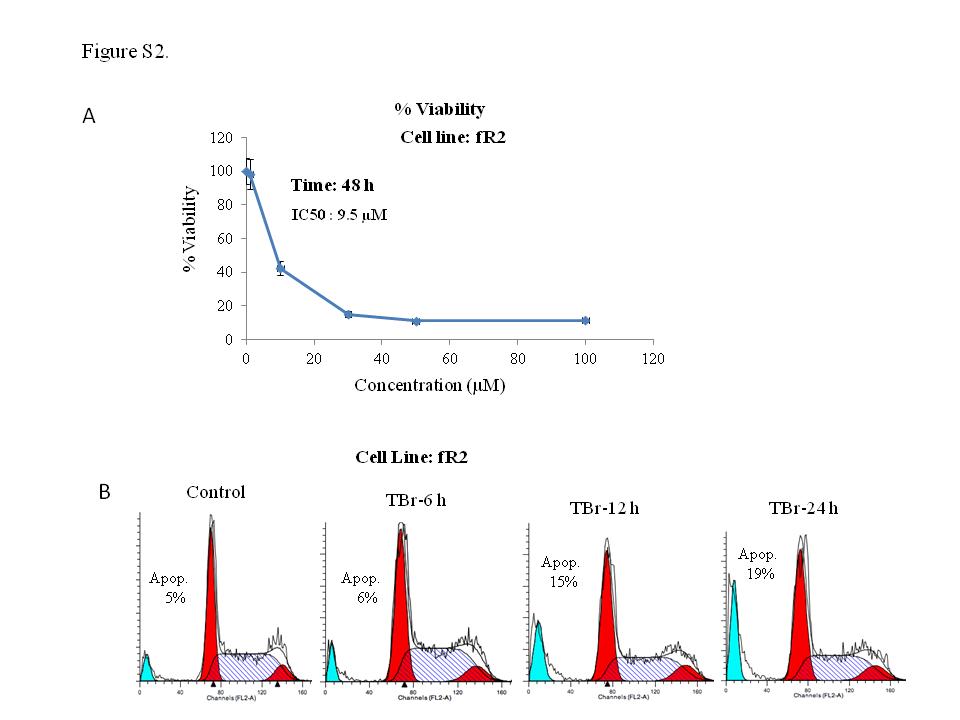

Supplement: Figure S2 — Effect of TBr on normal human breast epithelial cell line fR2. (A) MTT assay of TBr treated breast epithelial cell line fR2 for 48h (B) Cell cycle analysis of TBr (3 µM) treated fR2 cell line for 24h. (TIF) [file pone.0110411.s002.tif]

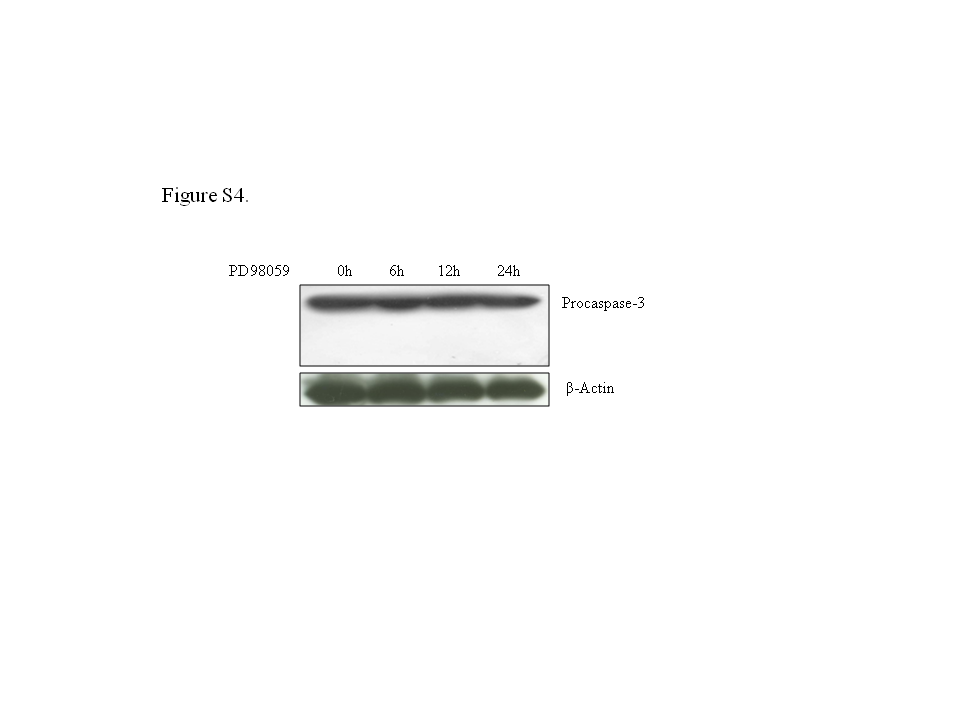

Supplement: Figure S4 — Effect of PD98059 on Caspase-3 and PARP-1 cleavage in HL-60 cells. Cells were treated with PD98059 (30 µM) for 24h followed by protein lysates preparation, SDS PAGE and immunoblotting of Caspase-3 and PARP-1. β actin was used as a positive control. (TIF) [file pone.0110411.s004.tif]
